# Mechanisms of the Indirect Effects of CMV Infection in Solid Organ Transplant Recipients: A Narrative Review

**DOI:** 10.3390/jcm15124671

**Published:** 2026-06-16

**Authors:** Anna Podraza, Dominika Dęborska-Materkowska, Dorota Kamińska, Krzysztof Mucha

**Affiliations:** 1 Department of Immunology, Transplantology, Nephrology and Internal Diseases, Warsaw Medical University, Nowogrodzka Str. 59, 02-006 Warsaw, Poland; ania.podraza001@gmail.com (A.P.); krzysztof.mucha@wum.edu.pl (K.M.); 2 Clinical Trials Support Centre, 4th Military Clinical Hospital, 50-981 Wrocław, Poland; dorotakaminska@interia.pl; 3 Division of Nephrology, Transplantology and Clinical Immunology, 4th Military Clinical Hospital, Faculty of Medicine, Wroclaw University of Science and Technology, 50-370 Wrocław, Poland; 4 Institute of Biochemistry and Biophysics, Polish Academy of Sciences, 02-106 Warsaw, Poland

**Keywords:** cytomegalovirus, solid organ transplantation, immune dysregulation, allograft rejection, endothelial dysfunction, opportunistic infections

## Abstract

Cytomegalovirus (CMV) is a major determinant of post-transplant morbidity in solid organ transplant recipients, not only through direct viral disease but also through a broad spectrum of indirect effects that may adversely influence graft and patient outcomes. This review summarizes current clinical and mechanistic evidence regarding the mechanisms of CMV-associated indirect injury in transplantation, drawing on human observational studies together with supporting in vitro and animal-model data. CMV establishes lifelong latency with intermittent reactivation and exerts sustained immunomodulatory effects on both innate and adaptive immunity, which may persist even during low-level viral replication. The mechanisms discussed include monocyte reprogramming, altered antigen presentation, T-cell and natural killer cell dysregulation, endothelial activation and dysfunction, chronic inflammatory signaling, impaired antimicrobial defense, and disturbances in metabolic regulation. The review considers how these mechanisms have been proposed to translate into major post-transplant complications, including acute rejection, chronic allograft dysfunction, cardiovascular and thrombotic disease, post-transplant diabetes, and increased susceptibility to secondary bacterial, fungal, and viral infections. It also addresses current preventive strategies, although evidence regarding their effectiveness in reducing indirect clinical outcomes remains limited and largely observational. Much of the supporting evidence is associative, and the contribution of CMV is often difficult to separate from that of the overall immunosuppressive burden and the comorbidities of transplant recipients. With these considerations, the available evidence supports regarding CMV not merely as an opportunistic pathogen, but as a persistent immunobiological driver of long-term transplant injury. Improved understanding of these indirect effects may enhance risk stratification, support biomarker-guided prevention, and inform future strategies aimed at reducing long-term graft dysfunction and patient morbidity after transplantation.

## 1. Introduction

Despite substantial advances in immunosuppressive strategies and antiviral prophylaxis, infectious complications remain a major determinant of long-term outcomes following solid organ transplantation (SOT). Among these, cytomegalovirus (CMV) is of particular clinical importance due to the high prevalence and capacity to influence graft and patient outcomes beyond direct viral disease [1,2]. Recent evidence indicates that cumulative CMV burden, including both total viral load exposure and the duration of viremia, is one of key determinants of long-term transplantation outcomes. In a large kidney transplant cohort, the predicted risk of graft failure and death was strongly associated with the cumulative duration of viremia and the overall viral load burden, with graft survival falling to 30% and patient survival to 7% among those with the highest levels of cumulative viremia [3]. The risk of CMV infection and disease is highest during the first months after transplantation, particularly during periods of intense immunosuppression and following the withdrawal of antiviral prophylaxis [1,4].

CMV infection manifestation can range from a systemic viral syndrome characterized by fever, malaise, cytopenias, and mild hepatic transaminitis, to tissue-invasive disease most commonly of the gastrointestinal tract, but virtually any organ may be affected [4,5]. However, the clinical impact of CMV in transplantation extends beyond overt viral disease. Accumulating evidence indicates that CMV exerts a broad range of indirect effects. CMV has emerged as a central immunomodulatory pathogen in transplantation [6]. Through latency and intermittent reactivation, CMV establishes a persistent interaction with the host immune system, reshaping both innate and adaptive immune response [7]. Indirect effects occur independently of high viral load and are the result of the virus altering the host immune response even during sustained low-level CMV replication [8]. This results in a complex dual effect characterized by sustained inflammatory activation alongside impaired antimicrobial defense. These mechanisms provide a biological framework linking CMV infection with acute rejection, chronic allograft dysfunction, cardiovascular diseases, post-transplant metabolic complications, and increased susceptibility to opportunistic infections [1,5,9]. These effects may occur even in the absence of clinically apparent viral disease and can significantly contribute to graft dysfunction, rejection, and long-term morbidity [1,10,11].

The risk of CMV infection and the magnitude of its indirect consequences are strongly influenced by donor and recipient serostatus at the time of transplantation. Four serostatus combinations are recognized: donor-seronegative/recipient-seronegative (D−/R−), donor-seropositive/recipient-seronegative (D+/R−), donor-seronegative/recipient-seropositive (D−/R+), and donor-seropositive/recipient-seropositive (D+/R+). The D+/R− combination confers the highest risk because the recipient lacks pre-existing CMV-specific immunity and may develop primary donor-derived infection under intense immunosuppression, often with higher-level DNAemia and a stronger inflammatory response [1]. By contrast, CMV-seropositive recipients harbor latent virus before transplantation and retain partial CMV-specific immune memory, which generally attenuates the severity of clinically apparent disease. However, this immunity may be weakened by post-transplant immunosuppression, allowing intermittent, low-level, or clinically silent reactivation even in the absence of overt CMV disease [12,13,14]. Such subclinical viral activity is unlikely to be immunologically inert: persistent or recurrent antigen exposure may sustain T-cell activation, promote expansion of highly differentiated effector-memory T-cell populations, contribute to immune senescence, and reprogram innate immune responses—alterations that may persist long after viral replication is controlled and continue to contribute to endothelial activation, intragraft inflammation, and alloimmune priming in R+ recipients [13,14].

Large registry-based studies support a graded serostatus-dependent model of CMV-related risk. In a paired kidney analysis of 9134 deceased-donor transplant recipients, D+/R− status was associated with higher all-cause mortality and infection-associated mortality compared with D+/R+ recipients receiving organs from the same donor, suggesting that the excess risk is not explained by donor organ factors alone [15]. Similarly, in a registry analysis of 54,078 adult liver transplant recipients, D+/R− serostatus was independently associated with increased graft loss and mortality, whereas other serostatus groups did not remain independently associated after adjustment; the persistence of this association beyond the first post-transplant year supports a contribution of mechanisms other than short-term clinically apparent CMV disease [16].

Although the clinical risk is generally lower in R+ recipients than in D+/R− recipients, reactivation may still induce durable immune alterations.

Clinical evidence supports that CMV reactivation in R+ recipients carries meaningful indirect graft consequences beyond the period of active viral replication. In a cohort study of kidney transplant recipients, late-onset CMV infection—occurring predominantly after prophylaxis cessation in R+ recipients with insufficient CMV-specific immune reconstitution—was independently associated with a considerably increased risk of allograft failure, underscoring that recipient seropositivity should not be equated with protection from indirect CMV-mediated graft injury [17].

In kidney transplant recipients with CMV DNAemia, longitudinal transcriptomic analysis demonstrated early activation of interferon signaling, cytotoxic T-cell pathways, macrophage-related pathways, CCR5-mediated immune-cell trafficking, and chemokine signaling, with several innate and adaptive immune pathways remaining altered after resolution of detectable DNAemia. Because most DNAemic patients in that cohort were CMV-seropositive, these findings support the concept that CMV reactivation in CMV+ recipients can have persistent immunologic consequences. This suggests that CMV-related immune alterations may extend beyond the period of detectable viral replication and may contribute to indirect effects in both primary infection and reactivation, although their intensity and biological profile are likely to differ between these settings [18].

The indirect effects discussed in this review should be understood as operating across both primary infection in seronegative recipients and reactivation or reinfection in seropositive recipients, with quantitative differences reflecting viral burden, timing of replication, donor-derived exposure, intensity of immunosuppression, and the degree of pre-existing CMV-specific immunity.

This review synthesizes current clinical and mechanistic evidence on the indirect effects of CMV in SOT recipients. Particular emphasis is placed on how virus-driven immune reprogramming, endothelial injury, metabolic alterations, and host–virus interactions converge to influence graft survival and patient outcomes. Understanding these mechanisms is essential for improving risk stratification and developing more targeted strategies to mitigate long-term complications in transplant recipients.

This article is a narrative (non-systematic) review. Relevant publications were identified through searches of PubMed/MEDLINE up to early 2026, using combinations of the terms “cytomegalovirus,” “CMV,” “solid organ transplantation,” “indirect effects,” “allograft rejection,” “cardiovascular disease,” “post-transplant diabetes,” “secondary infection,” “prophylaxis,” and “preemptive therapy,” supplemented by manual screening of reference lists and relevant international guidelines. Because this is a narrative review intended to integrate clinical and mechanistic perspectives rather than to provide a quantitative synthesis, no formal systematic-review or meta-analytic methodology was applied.

It should be emphasized that, whereas the epidemiological association between CMV and described indirect effects derives largely from human observational and registry studies, much of the mechanistic detail outlined below has been established in in vitro systems (infected endothelial cells, monocytes/macrophages, and vascular smooth muscle cells) and in animal models, and has not been directly demonstrated in human transplant tissue. These mechanistic pathways should therefore be regarded as biologically plausible contributors rather than proven causal mediators in transplant recipients.

## 2. Risk of Graft Rejection and Injury

Although modern immunosuppression has reduced incidence of acute rejection, the resulting infectious burden has become increasingly important for long-term graft survival. Cytomegalovirus (CMV) across SOT recipients is a key example, capable of inducing inflammatory programs that favor rejection.

Multiple clinical studies and large population-based analyses demonstrate that CMV infection is a significant risk factor for acute rejection across multiple transplanted organs. In a French national cohort of 20,473 solid organ transplant recipients, CMV disease was independently associated with an increased risk of graft rejection, regardless of timing after transplantation (odds ratio range 1.43–1.61) [11]. A recent systematic review and meta-analysis including 27 studies and 6308 SOT recipients showed that CMV infection approximately doubled the overall risk of acute rejection (pooled OR 2.02), with marked heterogeneity by organ type. The strongest association was observed in liver transplantation (OR 4.43), followed by kidney (OR 2.22) and lung transplantation (OR 1.38), while no statistically significant association was detected in heart transplant recipients [2].

Beyond acute rejection, CMV infection and replication have been linked to chronic allograft dysfunction and graft loss. Long-term observational study of renal transplant recipients demonstrates that CMV viremia persisting or recurring in the chronic phase (>1 year post-transplant) is significantly associated with chronic rejection and subsequent graft loss, even when CMV infection is asymptomatic [19]. CMV is also associated with organ-specific graft injury across transplantation, including accelerated coronary vasculopathy in cardiac transplantation and bronchiolitis obliterans syndrome in lung transplant recipients [6].

While clinical and experimental data clearly link CMV infection to allograft rejection and dysfunction, the immunological mechanisms responsible for these immunostimulatory effects are ongoing fields of research.

After primary infection, CMV establishes lifelong latency with intermittent reactivation, requiring ongoing immune surveillance to maintain control of viral replication [12,13,14]. More than half of CMV’s genes help the virus evade the immune system targeting adaptive and innate immunity [12,20]. This persistent interaction can remodel local graft immunity by shaping effector cell responses, inflammatory signaling, and antigen presentation, thereby lowering the threshold for alloimmune activation [14]. Below, the main immunological and cellular mechanisms proposed to link CMV with allograft rejection and dysfunction are summarized.

Monocytes are one of the primary cellular targets for CMV, and CMV infection profoundly alters monocyte function in ways that promote allograft rejection [6,12,14]. Transcriptomic and functional analyses show that CMV-infected monocytes upregulate antiviral and pro-inflammatory programs strongly associated with rejection, including interferon-stimulated genes and inflammasome-related pathways. CMV infection induces increased expression of cytokines and chemokines that facilitate immune cell recruitment into the graft, and many of which have been implicated as biomarkers of graft injury and rejection. At the same time, CMV induces downregulation of key innate immune regulatory and scavenger receptor pathways, disrupting immune homeostasis and further tipping the balance toward alloimmune activation. These CMV-driven transcriptional changes amplify alloimmune responses, lower the threshold for acute rejection, and may contribute to the development of allograft dysfunction [6].

In kidney transplantation, antibody-dependent cell-mediated cytotoxicity pathways involving NK cells have been highlighted to be active during antibody-mediated rejection and consistently suggest mediation of allograft injury in a complement-independent manner [21]. CMV infection results in a higher overall NK cell alloreactivity, which augments antibody-dependent reactivity, including reactivity to anti–HLA-specific antibodies, which may pose an additional adverse effect on graft survival [22].

In a murine renal allograft model, CMV infection was associated with increased ingraft infiltration of Th17 cells expressing IL-17A and IFN-γ and/or TNF-α, thereby promoting a highly inflammatory ingraft immune profile. Th17 cell accumulation correlates positively with Th1 cell infiltration and inversely with regulatory T cell (Treg) frequencies, thereby shifting the immune balance toward rejection. Consistent with these findings, elevated IL-17A serum levels have been observed during acute rejection episodes in renal transplant recipients with CMV DNAemia, supporting a direct link between CMV-driven inflammation and alloimmune-mediated graft injury [23].

CMV infection generates large populations of effector and memory T cells that persist long term and can directly contribute to allograft rejection through cross-reactive reactions [20,24]. In a clinical and molecular study of kidney transplant recipients, pre-transplant CMV-specific effector/memory T-cell responses are strongly associated with subsequent acute rejection and inferior graft function. Using T-cell receptor repertoire sequencing, identical TCR-β clonotypes were identified among CMV-reactive and donor alloantigen-reactive T cells, providing direct evidence of functional cross-reactivity. Importantly, these shared clonotypes were also detected within renal allograft biopsies, particularly in patients with concurrent CMV DNAemia and rejection. CMV reactivation was associated with expansion of these cross-reactive T-cell populations, indicating that viral antigenic stimulation can directly amplify alloimmune responses. Together, these findings demonstrate that CMV can promote rejection through direct T-cell cross-reactivity with donor antigens, in addition to its indirect pro-inflammatory effects [24].

As shown in experimental infection models, CMV modulates antigen presentation by altering the expression of both MHC class I and class II molecules, with important implications for alloimmune activation in transplantation [13,25,26]. CMV disrupts multiple steps of the MHC-I antigen presentation pathway, downregulates MHC class I on infected cells, limiting direct CD8^+^ T-cell recognition [13], while simultaneously promoting increased MHC class I expression on surrounding uninfected bystander cells in response to local inflammatory signals. This pattern may sustain immune activation within the tissue despite effective viral immune evasion [25,27]. In parallel, in vitro studies in a myeloid cell line indicate that CMV suppresses MHC class II expression in infected monocytes, impairing CD4^+^ T-cell recognition of infected cell [14,26]. By contrast, CMV infection can induce MHC class II expression on endothelial cells, potentially enhancing alloimmune recognition of the graft [27]. Together, these effects reshape ingraft antigen presentation in a manner that favors inflammation and lowers the threshold for allograft rejection. CMV infection is also linked to increased expression of endothelial adhesion molecules and their leukocyte ligands, and the development of endothelitis and anti-endothelial cell antibodies, which results in further graft injury [12,27,28,29].

Clinically, CMV replication frequently necessitates a reduction in immunosuppressive therapy to facilitate immune control of viral replication. This reduction, although always required, can independently increase the risk of acute rejection and may further amplify CMV-driven alloimmune responses [1,27,30].

**Figure 1:**
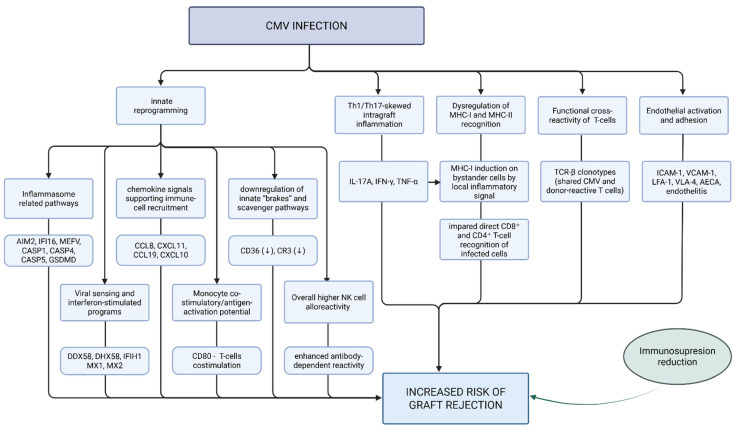
Proposed mechanisms by which CMV infection promotes immune dysregulation and increases the risk of graft rejection. CMV infection induces coordinated innate and adaptive immune dysregulation, including innate immune reprogramming, altered antigen presentation, T-cell dysfunction, and endothelial activation, which together promote intragraft inflammation and increase the risk of graft rejection. AECA—anti-endothelial cell antibodies; AIM2—absent in melanoma 2; C3—complement component 3; CASP1/4/5—caspase 1/4/5; CCL—C–C motif chemokine ligand; CD—cluster of differentiation; CR3—complement receptor 3; CXCL—C–X–C motif chemokine ligand; DDX58 (RIG-I)—DEAD-box helicase 58; DHX58—DExH-box helicase 58; GSDMD—gasdermin D; ICAM-1—intercellular adhesion molecule 1; IFI16—interferon gamma inducible protein 16; IFIH1 (MDA5)—interferon induced with helicase C domain 1; IFN-γ—interferon gamma; IL—interleukin; LFA-1—lymphocyte function-associated antigen 1; MEFV—Mediterranean fever gene (pyrin); MHC—major histocompatibility complex; MX1/MX2—myxovirus resistance proteins 1 and 2; NK—natural killer (cell); TCR—T-cell receptor; TCR-β—T-cell receptor beta chain; Th1/Th17—T helper type 1/type 17; TNF-α—tumor necrosis factor alpha; VCAM-1—vascular cell adhesion molecule 1; VLA-4—very late antigen-4.

As summarized in Figure 1, CMV infection is proposed to orchestrate a multilayered immunologic program involving innate immune reprogramming, Th1/Th17-dominant ingraft inflammation, heterologous T-cell cross-reactivity, reshaping of antigen presentation, endothelial activation, and clinically driven immunosuppression modulation—converge to amplify alloimmune responses, lower the threshold for acute rejection, and promote chronic allograft dysfunction in the setting of CMV infection.

## 3. CMV Infection and Increased Cardiovascular Risk

Beyond its effects on graft function and opportunistic infections, CMV infection has been recognized as an important contributor to the development and progression of cardiovascular disease [31,32]. CMV has been detected in atherosclerotic plaques and vascular tissues, and viral persistence with episodic reactivation may contribute to chronic vascular inflammation and plaque progression [31].

In transplant populations, a recent systematic review and meta-analysis of 12 studies demonstrated that CMV infection among abdominal organ transplant recipients is associated with an approximately doubling of the risk of cardiovascular events (adjusted HR = 2.17), including myocardial infarction, stroke, heart failure, peripheral arterial disease, and cardiovascular mortality [32]. In addition, a comparative study of renal transplant recipients and healthy adults demonstrated that CMV antibody levels were significantly higher in transplant recipients and independently associated with impaired endothelial function, measured by reduced flow-mediated dilation (FMD) [33]. Consistent with these findings, clinical evidence in non-transplant populations further supports the link between CMV infection and endothelial dysfunction. In patients with ST-elevation myocardial infarction (STEMI), productive CMV infection was associated with significantly impaired endothelial function, as assessed by FMD. Notably, this relationship was independent of traditional cardiovascular risk factors, suggesting a direct contribution of CMV to endothelial injury and vascular dysfunction [34].

In parallel, CMV exposure has also been strongly associated with an increased risk of arterial and venous thromboembolic events following transplantation. In a large cohort of CMV-seronegative non-thoracic SOT recipients, who received an organ from either a seropositive (D+/R−) or seronegative (D−/R−) donor. The D+/R− group showed a substantially increased risk of post-transplant thrombotic events, including deep venous thrombosis, pulmonary embolism, and arterial thromboses. Importantly, this association was observed even in the absence of active CMV infection at the time of thrombosis, indicating that thrombotic risk represents an indirect and sustained effect of CMV exposure rather than a direct consequence of viral replication [35].

**Figure 2:**
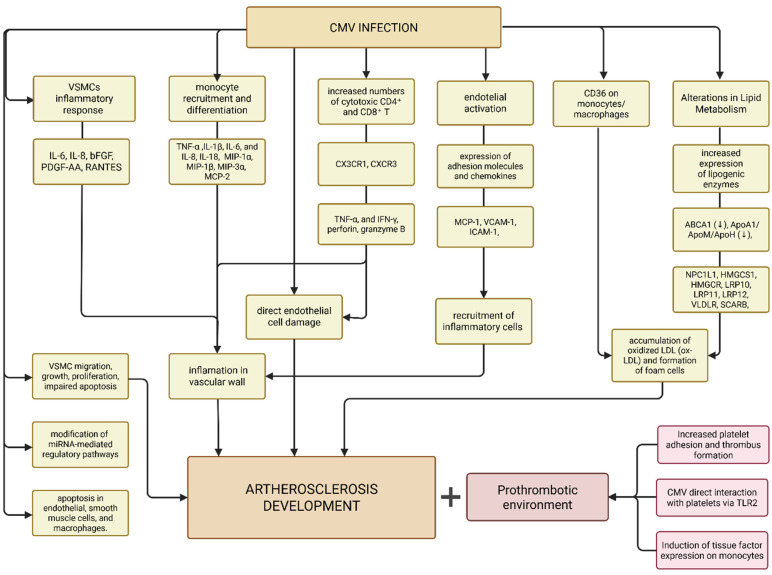
Proposed mechanisms by which CMV infection promotes atherosclerosis development and a prothrombotic state. CMV infection promotes atherosclerosis through coordinated effects on vascular inflammation, immune activation, and lipid metabolism, including monocyte recruitment, endothelial activation, cytotoxic T-cell responses, and vascular smooth muscle cell dysfunction. These processes enhance inflammatory cell infiltration, endothelial injury, and accumulation of oxidized LDL with foam cell formation. In parallel, CMV induces a prothrombotic state via platelet activation, interaction with platelets through TLR2, and increased tissue factor expression on monocytes. ABCA1—ATP-binding cassette transporter A1; ApoA1—apolipoprotein A1; ApoM—apolipoprotein M; ApoH—apolipoprotein H; bFGF—basic fibroblast growth factor; CD—cluster of differentiation; CX3CR1—C–X3–C motif chemokine receptor 1; CXCR3—C–X–C motif chemokine receptor 3; HMGCS1—3-hydroxy-3-methylglutaryl-CoA synthase 1; HMGCR—3-hydroxy-3-methylglutaryl-CoA reductase; ICAM-1—intercellular adhesion molecule 1; IL—interleukin; LRP11—low-density lipoprotein receptor-related protein 11; LRP12—low-density lipoprotein receptor-related protein 12; MCP-1—monocyte chemoattractant protein 1; MIP-1α/β—macrophage inflammatory protein 1 alpha/beta; MIP-3α—macrophage inflammatory protein 3 alpha; NPC1L1—Niemann-Pick C1-like 1; ox-LDL—oxidized low-density lipoprotein; PDGF-AA—platelet-derived growth factor AA; RANTES—regulated upon activation, normal T cell expressed and secreted; SCARB—scavenger receptor class B; TLR2—Toll-like receptor 2; TNF-α—tumor necrosis factor alpha; VCAM-1—vascular cell adhesion molecule 1; VLDLR—very low-density lipoprotein receptor; VSMCs—vascular smooth muscle cells.

CMV appears to influence several biological pathways central to atherogenesis, which are discussed below and illustrated in Figure 2.

The virus can infect multiple cell types involved in vascular pathology, including endothelial cells, macrophages, dendritic cells, fibroblasts, and vascular smooth muscle cells. Through persistent infection and episodic reactivation, CMV may sustain chronic inflammatory and immune responses within the vascular wall, thereby contributing to plaque development and progression [36]. CMV infection is also associated with persistent systemic inflammation, characterized by elevated circulating cytokines which are independently linked to cardiovascular risk [37].

Infected endothelial cells upregulate adhesion molecules and chemokines, facilitating recruitment of inflammatory cells to the vascular wall and promoting their adhesion and transmigration [36,37]. In addition, CMV infection can induce endothelial apoptosis and direct endothelial injury, further disrupting vascular barrier integrity and promoting early atherogenic changes [31]. CMV-associated endothelial dysfunction is further linked to downregulation of endothelial nitric oxide synthase, contributing to impaired vasodilation and vascular dysfunction [37]. CMV-mediated impairment of endothelial cyclic guanosine monophosphate (cGMP) levels reflects reduced bioavailability of nitric oxide. Consistently, transplant recipients with active CMV infection exhibit elevated plasma levels of asymmetric dimethylarginine (ADMA), which is associated with an increased risk of transplant arteriosclerosis [38].

The replication of cytomegalovirus in endothelial cells and macrophages —as demonstrated in vitro—triggers the release of adhesion molecules, proinflammatory cytokines, and matrix metalloproteinases, as well as cellular death [39,40]. Circulating monocytes are recruited to inflamed vascular tissues, where they differentiate into macrophages and participate in plaque formation [36,37]. CMV infection enhances monocyte survival, adhesion to endothelial cells, and transendothelial migration, leading to increased macrophage accumulation within the vascular wall [36]. This process is further facilitated by CMV-induced impairment of endothelial barrier function, enhancing monocyte infiltration into the vascular intima [41].

In addition, CMV infection may alter lipid metabolism by increasing the expression of molecules involved in cholesterol uptake and lipid synthesis while downregulating pathways responsible for cholesterol efflux [36,41]. Together with macrophage secretion of pro-inflammatory cytokines CMV infection upregulates the scavenger receptor CD36 and enhances oxidized LDL uptake in monocyte/macrophage cell lines [36,37]. These alterations favor intracellular accumulation of oxidized LDL and the formation of lipid-laden foam cells, a hallmark of early atherosclerotic lesions [31,36].

During atherosclerotic lesion development, inflammatory cytokines promote the recruitment of circulating T cells to the vascular wall. CMV infection further reshapes the T-cell compartment by expanding highly differentiated cytotoxic T cells that are preferentially attracted to activated endothelium, thereby facilitating their accumulation at sites of vascular inflammation and injury [36,37]. Increased numbers of CMV-specific CD4^+^ and CD8^+^ T cells accumulate within vascular tissues and exhibit strong cytotoxic activity through molecules such as granzyme B and perforin, enabling direct endothelial injury [36]. In addition, CMV infection promotes the accumulation of senescent T-cell populations that produce elevated levels of pro-inflammatory cytokines, particularly interferon-γ [36]. Notably, CMV-specific T cells have been identified within atherosclerotic plaques and may cross-react with vascular antigens, suggesting a role for molecular mimicry in sustaining vascular inflammation [41].

Infected vascular smooth muscle cells show increased migration from the vascular media to the intima, enhanced proliferation, and resistance to apoptosis, contributing to intimal thickening and plaque formation. CMV-infected vascular smooth muscle cells also produce inflammatory mediators and growth factors that promote vascular inflammation and extracellular matrix remodeling, processes associated with plaque progression and destabilization [36]. In addition, viral proteins such as US28 and CMV-derived IL-10 homologs enhance chemokine signaling and immune cell recruitment, further promoting plaque progression and instability [41].

Finally, CMV infection may regulate vascular pathology through viral proteins and microRNAs that modulate host signaling pathways. These factors influence cellular processes involved in inflammation, angiogenesis, apoptosis, and vascular remodeling, contributing to the development and progression of atherosclerotic lesions [36,41].

CMV infection also contributes to a prothrombotic vascular environment. Endothelial activation and inflammation promote platelet adhesion and increase the risk of thrombus formation. In addition, CMV-driven immune activation, particularly via TNF-producing T cells, can induce tissue factor expression on monocytes, linking inflammation with coagulation pathways and further enhancing cardiovascular risk [37]. CMV can directly interact with platelets via TLR2, inducing release of proinflammatory mediators such as CD40L, IL-1β, and VEGF, amplifying thrombosis and vascular inflammation [31].

Taken together, these mechanisms provide a mechanistic link between CMV infection and vascular pathology, which may be particularly relevant in immunocompromised populations, including solid organ transplant recipients, where CMV reactivation and immune dysregulation are common.

## 4. CMV as a Risk Factor for Post-Transplant Diabetes

Diabetes mellitus has long been linked to viral infections, with evidence that viral infections may accompany or precede disease onset and contribute to immune pathways involved in β-cell injury [42]. In transplant populations, infection—including CMV—is repeatedly listed among transplant-specific risk factors for post-transplant diabetes, alongside traditional type-2-diabetes risks (age, obesity, family history) and drug effects (notably steroids and calcineurin inhibitors) [43,44,45,46,47].

Multiple observational studies support association between CMV infection and post-transplant diabetes. A prospective cohort study reported that CMV infection in SOT recipients was associated with a higher incidence of early post-transplant diabetes (26% in CMV-infected vs. 6% in controls) and remained an independent predictor in multivariable analysis [48]. Consistent findings have been reported in a single-center retrospective cohort study of 284 kidney transplant recipients. Post-transplant CMV infection was more common among those who developed post-transplant diabetes (56% vs. 40%) and was associated with approximately 2.3-fold higher odds of post-transplant diabetes [49]. In a larger cohort of 1040 kidney transplant recipients with long-term follow-up (median 12.7 years), diabetes developed in 28.5% of patients. CMV infection occurred significantly more often in recipients who developed diabetes than in those who did not. The authors identified CMV infection, alongside others, as a factor associated with increased PTDM risk [50].

In a review with meta-analysis, Einollahi et al. concluded that CMV infection is associated with a higher risk of posttransplant diabetes, with pooled analyses suggesting an approximately twofold increase in risk across seven cohort studies. The authors also emphasize that heterogeneity in CMV definitions/diagnostics, post-transplant diabetes criteria and immunosuppression protocols likely contributes to variation across individual studies [51]. Conversely, some studies suggest that CMV might not be an independent risk factor for post-transplant diabetes [52,53]. A systematic review and meta-analysis of 24 case–control studies (7140 kidney transplant recipients) found no significant association between CMV infection and post-transplant diabetes. The authors noted that CMV effect may be inconsistent across cohorts and potentially outweighed by stronger determinants such as age, BMI, tacrolimus exposure, acute rejection, and hepatitis infections [52].

An important but insufficiently explored distinction is whether the metabolic risk attributed to CMV reflects active post-transplant viral replication or pre-transplant seropositivity. Most of the studies discussed above define CMV exposure as active infection. However, other evidence suggests that baseline serostatus itself may also carry metabolic relevance, even in the absence of detectable post-transplant replication. In a kidney transplant cohort managed with universal prophylaxis, in which post-transplant viremia was uncommon, pre-transplant CMV IgG seropositivity was significantly more frequent among recipients who developed new-onset diabetes [54]. This observation is consistent with data from non-transplant populations: in a prospective cohort, CMV seropositivity was independently associated with diabetes-related mortality [55], while in the Leiden 85-plus study, CMV seropositivity correlated with type 2 diabetes and higher HbA1c levels [56]. Comprehensive analyses that evaluate recipient and donor CMV serostatus separately [57], together with observations that viral serology-guided immunosuppression may influence diabetes incidence in transplant patients [46], further support the need to distinguish baseline CMV exposure from active post-transplant replication.

If pre-transplant CMV seropositivity independently contributes to PTDM risk, baseline serology could improve metabolic risk stratification before transplantation. Conversely, if active CMV replication is the dominant driver, prevention of post-transplant DNAemia or CMV disease would represent the more relevant intervention. Prospective studies directly comparing PTDM incidence among seropositive recipients with and without post-transplant CMV DNAemia are needed to clarify the relative contribution of these two exposure states.

**Figure 3:**
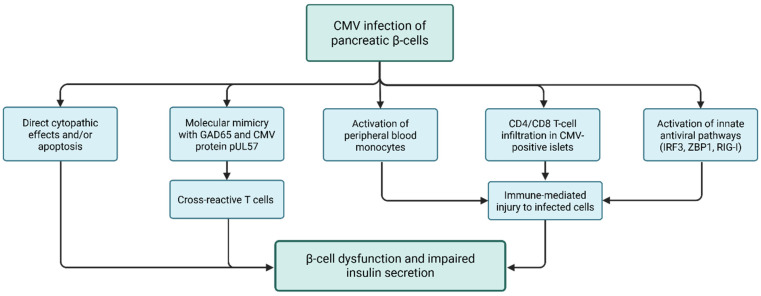
Proposed mechanisms by which CMV infection contributes to pancreatic β-cell dysfunction and impaired insulin secretion. CMV infection of pancreatic β-cells induces direct cytopathic effects and immune-mediated injury through activation of innate antiviral pathways, and infiltration of CD4^+^ and CD8^+^ T cells. In addition, molecular mimicry and cross-reactive T-cell responses further contribute to β-cell damage, resulting in impaired insulin secretion. CMV—cytomegalovirus; GAD65—glutamic acid decarboxylase 65; IRF3—interferon regulatory factor 3; pUL57—CMV phosphoprotein UL57; RIG-I—retinoic acid-inducible gene I; ZBP1—Z-DNA-binding protein 1.

Accumulating experimental and clinical evidence suggests that cytomegalovirus (CMV) may contribute to post-transplant diabetes through overlapping direct and immune-mediated pathways that lead to β-cell dysfunction and impaired insulin secretion [58]. The proposed mechanisms are illustrated in Figure 3. CMV may injure pancreatic β-cells through direct infection of endocrine tissue, with cytopathic effects and/or apoptosis as plausible mechanisms of β-cell loss [58]. Infected endocrine cells may also become targets of immune-mediated injury, including killing by NK cells and CMV-specific cytotoxic T cells. In addition, molecular mimicry has been proposed, as GAD65 shares sequence similarity with the CMV protein pUL57, which may enable cross-reactive T cells and amplify autoimmune β-cell injury [58]. In vitro studies in human pancreatic endocrine cells further show that CMV infection can increase endocrine-cell immunogenicity, marked by upregulation of MHC class I and ICAM-1 and induction of pro-inflammatory cytokine release, together with enhanced activation of peripheral blood monocytes [59]. Consistent with this, human tissue data demonstrate that CMV-positive pancreatic islets exhibit macrophage and CD4/CD8 T-cell infiltration and activation of innate antiviral pathways, supporting a model of locally amplified innate and adaptive immune activation contributing to β-cell injury [60].

Importantly, the contribution of CMV may be particularly relevant during the first months after transplantation, when the risk of CMV replication is highest and coincides with exposure to high doses of glucocorticoids and calcineurin inhibitors—both of which exert well-established toxic effects on pancreatic β-cells and impair insulin secretion—while glucocorticoids primarily drive insulin resistance with secondary β-cell impairment, calcineurin inhibitors cause direct β-cell toxicity with reduced insulin secretion [61,62,63,64].

## 5. CMV and Risk of Secondary Infections in SOT

While CMV infection drives sustained pro-inflammatory immune activation, it simultaneously alters immune regulation in ways that suppress antimicrobial defenses and increase susceptibility to secondary infections [7].

Clinical data from transplant studies included in international guidelines support a contributory role of CMV in secondary infections, as prevention strategies targeting CMV infection have been associated with a reduction in rates of opportunistic infections (and other indirect effects) indicating that CMV-driven immunosuppression actively promotes infection risk [1].

CMV infection is associated with a markedly increased risk of opportunistic infections, particularly within the first months following CMV diagnosis. In a matched case–control analysis, CMV-infected patients exhibited a 5.6-fold within 90 days and 4.3-fold within 180 days higher risk of opportunistic infections compared with CMV-negative controls, with fungal infections accounting for the majority of cases. Importantly, the development of opportunistic infection with CMV infection was associated with significantly increased mortality [65].

CMV infection is strongly associated with concurrent and sequential viral reactivations in solid organ transplant recipients. A comprehensive review of multiviral infections in transplantation reports that reactivation of two or more viruses occurs in up to 89% of SOT recipients, with CMV most frequently co-reactivating with Epstein–Barr virus, BK virus, and other human herpesviruses [66]. CMV donor seropositivity and donor–recipient serologic mismatch have been identified as risk factors for viral coinfection, indicating an increased susceptibility to viral reactivation in this setting [66].

Beyond viral coinfections, CMV infection is also associated with an increased risk of bacterial secondary infections. The spectrum of bacterial secondary infections includes both common and opportunistic pathogens, with clinical presentations such as bacteremia, pneumonia, urinary tract infections, and surgical site infections. In a large cohort study of kidney transplant recipients, bacterial coinfections were observed in approximately 38% of patients with CMV disease [4].

In the systematic review and meta-analysis CMV disease was consistently associated with an increased risk of early invasive fungal infections, with pooled analyses demonstrating an approximately three-fold increased risk across solid organ transplant populations [67]. Pneumocystis jirovecii pneumonia (PJP) represents a CMV-associated opportunistic fungal infection associated with substantial morbidity and mortality. A comprehensive systematic review and meta-analysis identified CMV infection or disease as a strong independent risk factor for PJP across solid organ transplant populations, conferring approximately a three-fold increased odds of disease [68]. Invasive aspergillosis represents another severe CMV-associated fungal complication in SOT recipients. A large systematic review and meta-analysis including over 5400 SOT recipients demonstrated that post-transplant CMV infection was associated with a more than threefold increased risk of subsequent invasive aspergillosis (pooled OR 3.31) [69].

**Figure 4:**
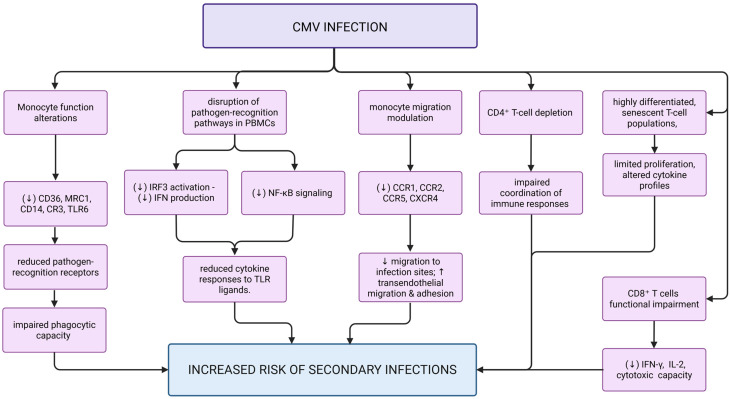
Proposed mechanisms by which CMV infection increases the risk of secondary infections CMV infection induces coordinated innate and adaptive immune dysfunction, including impaired monocyte function, disruption of pathogen-recognition pathways, altered cell migration, and lymphocyte dysfunction. These changes result in reduced pathogen recognition, blunted cytokine responses, impaired immune coordination, and defective effector function, ultimately increasing susceptibility to secondary infections. CCR—C–C chemokine receptor; CD—cluster of differentiation; CR3—complement receptor 3; CXCR4—C–X–C chemokine receptor 4; IFN—interferon; IFN-γ—interferon gamma; IL—interleukin; IRF3—interferon regulatory factor 3; MRC1—mannose receptor C-type 1; NF-κB—nuclear factor kappa-light-chain-enhancer of activated B cells; PBMCs—peripheral blood mononuclear cells; TLR—Toll-like receptor.

Mechanistic studies provide explanations for these clinical observations and proposed mechanisms are summarized in Figure 4.

CMV infection in SOT recipients induces profound immunomodulation through multiple mechanisms that impair both innate and adaptive immunity, rendering patients highly susceptible to secondary bacterial, fungal, and viral infections. Monocytes are key cellular targets of CMV and undergo profound functional alterations during infection [70]. CMV disrupts multiple core monocyte effector functions, including phagocytosis, antigen presentation, cytokine production, and migration [70]. CMV-infected monocytes exhibit impaired phagocytic capacity against pathogens, contributing to an increased risk of secondary infections. Transcriptomic and functional analyses demonstrate that CMV infection induces a profound reprogramming of innate immune pathways, characterized by upregulation of intracellular antiviral pattern recognition receptors alongside downregulation of surface receptors critical for bacterial and fungal recognition, resulting in diminished phagocytosis of opportunistic organisms [6]. TLRs are central components of the innate immune response and play a key role in the recognition and elimination of viral, bacterial, and fungal pathogens. Activation of TLRs normally induces downstream signaling pathways leading to the production of pro-inflammatory, antiviral, and regulatory cytokines, which are essential for coordinating effective immune responses. However, CMV infection is associated with blunted cytokine responses to bacterial, fungal, and viral pathogen-associated molecular patterns (PAMPs). In their study, L’Huillier et al. demonstrated that peripheral blood mononuclear cells (T cells, B cells, NK cells, monocytes) from SOT patients with CMV infection, when exposed to various Toll-like receptor (TLR) ligands representing bacterial, fungal, and viral pathogens, produce significantly reduced cytokine responses compared to uninfected patients, indicating a global dysregulation of innate immune signaling [71]. Importantly, this impairment was independent of CMV viral load, clinical presentation, leukocyte counts, and intensity of immunosuppression, supporting a direct CMV-specific immunomodulatory mechanism. Mechanistically, in vitro molecular studies have shown that CMV interferes with key antiviral including TLR-mediated signaling to evade innate immunity. It impairs interferon regulatory factor 3 (IRF3) activation, suppressing type I interferon production, and inhibits NF-κB signaling via viral proteins and microRNAs, thereby reducing pro-inflammatory and antiviral responses [72,73,74]. Collectively, these immune evasion mechanisms impair host responses to heterologous pathogens, thereby increasing susceptibility to secondary infections [71].

Furthermore, CMV alters monocyte migration dynamics by downregulating chemokine receptors, impairing directed migration toward sites of infection, while simultaneously enhancing transendothelial migration and adhesion to endothelial cells, facilitating viral dissemination [70].

CMV infection is associated with profound alterations in lymphocyte subsets and function, characterized by marked CD4^+^ T-cell depletion and impaired CD8^+^ T-cell effector responses. Solid organ transplant recipients with CMV infection exhibit significantly reduced CD4^+^ T-cell counts, leading to impaired coordination of immune responses and reduced activation of both innate and adaptive immune pathways. In parallel, CD8^+^ T cells demonstrate functional impairment despite preserved absolute counts, with decreased interferon-γ production, reduced IL-2 secretion and reduced cytotoxic degranulation capacity, reflecting defective antiviral effector function [75,76,77]. In addition, CMV drives the expansion of highly differentiated, senescent T-cell populations, which display limited proliferative capacity and altered cytokine profiles [75,78]. These findings suggest that immune cells are present but functionally impaired, what can contribute to increased susceptibility to opportunistic infections. In parallel, CMV infection disrupts antigen presentation through mechanisms described in Section 2 (downregulation of MHC class I and II, inhibition of dendritic cell differentiation, loss of co-stimulatory molecules CD40 and CD80), thereby further impairing adaptive immune activation [70].

## 6. Impact of Prophylaxis and Preemptive Therapy on Indirect Effects of CMV

Current CMV prevention strategies in solid organ transplantation are intended not only to reduce overt CMV infection and disease, but also to mitigate the broader indirect consequences of viral replication. Although both universal prophylaxis (routine antiviral administration to at-risk SOT recipients for a defined post-transplant period) and surveillance-based preemptive therapy (serial virologic monitoring with treatment initiated upon detection of viral replication) are accepted preventive approaches with major influence on overall patient and graft outcomes, their comparative impact on indirect CMV-related outcomes remains incompletely established [1,79].

Part of this uncertainty also reflects the difficulty of disentangling the contribution of CMV from the many other factors that influence indirect post-transplant outcomes. Contemporary management has shifted toward the earliest possible detection of viral replication and prompt initiation of treatment, supported by both universal prophylaxis and surveillance-based preemptive strategies and by increasingly sensitive molecular monitoring [1]. These approaches were developed and are recommended primarily to reduce the burden of CMV infection and disease [1,80,81]. A consequence of their widespread adoption is that overt, high-level, and prolonged CMV replication has become comparatively less common, so that much of the indirect effect now attributable to CMV may operate at the level of low-level or subclinical viral activity rather than clinically apparent disease [71]. Because contemporary cohorts are actively monitored and treated, the magnitude and duration of viral exposure are correspondingly limited, which may attenuate observed associations between CMV and indirect outcomes and complicate efforts to demonstrate them convincingly—a caveat that should be borne in mind when interpreting the comparative data discussed below.

Recent large systematic reviews and meta-analyses have consistently shown that, compared with preemptive therapy, prophylaxis more effectively reduces CMV infection and replication, but does not clearly improve CMV disease, all-cause mortality, or major indirect clinical outcomes such as acute rejection, graft loss, and bacterial or fungal infections [80,81]. Nevertheless, because during preemptive therapy CMV replication and low-level viremia occur more frequently than during prophylaxis, this strategy may be less effective in preventing indirect effects driven by early or subclinical viral activity. The current guidelines specifically note that preemptive therapy may not fully prevent indirect effects of CMV infection, including effects on graft and patient survival, and that the available evidence remains conflicting [1]. At the same time, preemptive therapy appears to be associated with a lower risk of late-onset CMV disease, possibly because low-level viral exposure may allow development of CMV-specific immunity, whereas prophylaxis may postpone this process [81].

This pattern is reinforced by randomized trials designed to examine also indirect outcomes. In the OVERT study, 140 kidney transplant recipients randomized to valganciclovir prophylaxis or preemptive therapy had a similar incidence of acute rejection at 12 months, despite significantly lower CMV DNAemia in the prophylaxis group (44% vs. 75%) [82]. Likewise, in the VIPP trial of CMV-seropositive (R+) renal recipients followed for up to 7 years, prophylaxis was substantially more effective than preemptive therapy in preventing CMV infection and disease, yet both strategies were equally effective in preventing graft loss and death [83]. These trials show directly that superior virological control does not necessarily translate into proportionally better indirect or hard clinical outcomes.

Several mechanisms may explain this apparent disconnect. Many indirect effects appear to be mediated by CMV-induced immune dysregulation that is not strictly proportional to the level of viral replication. Ex vivo studies have shown that innate immune responses to bacterial, fungal, and viral Toll-like receptor ligands are blunted during CMV infection independently of viral load, clinical presentation, or immunosuppression, indicating that simply lowering viral burden may not fully restore antimicrobial immune competence [71]. Antiviral prophylaxis, while suppressing replication, also delays the development of CMV-specific immunity: in D+/R− liver transplant recipients, preemptive therapy generated significantly greater CMV-specific T-cell, NK-cell, and neutralizing antibody responses than prophylaxis, with differences persisting to 12 months [84]. Because protective immune reconstitution is postponed, prophylaxis may shift viral activity—and its indirect consequences—to the post-prophylaxis period, manifesting as late-onset CMV disease and ongoing immunologic changes [81]. In addition, subclinical or low-level reactivation occurring below the threshold for clinical detection or treatment may continue to drive endothelial activation, immune senescence, and epigenetic reprogramming that persist after viral suppression [78]. Together, these observations indicate that the indirect effects of CMV might be driven by qualitative immune modulation and the timing of immune reconstitution, not solely by the quantity of detectable virus that current strategies are designed to suppress.

These mechanistic considerations have direct implications for prevention strategy, which current guidelines address in a risk-stratified, organ-specific manner [1]. The Fourth International Consensus Guidelines tailor the choice and duration of prophylaxis to donor/recipient serostatus and organ type, reserving the most intensive prevention for the highest-risk groups. For D+/R− kidney transplant recipients, extended prophylaxis (up to 200 days) is recommended, supported by the IMPACT trial, in which 200 days of valganciclovir significantly reduced confirmed CMV disease compared with 100 days (16.1% vs. 36.8% at 12 months), with the benefit sustained at 2 years (21.3% vs. 38.7%), indicating a genuine reduction in disease incidence rather than merely a delay in onset. Notably, this longer prophylaxis did not reduce biopsy-proven acute rejection (11% vs. 17%), reinforcing that improved control of direct CMV disease does not necessarily translate into better indirect outcomes [85,86]. The CAPSIL randomized trial further demonstrated that in D+/R− liver transplant recipients preemptive therapy was actually superior to prophylaxis for preventing CMV disease, illustrating that the optimal strategy is organ-specific rather than uniformly favoring longer or more intensive prophylaxis [1,87]. For lung transplant recipients—at high risk of both direct disease and indirect complications such as chronic lung allograft dysfunction—prolonged prophylaxis (commonly 6–12 months) is advised [1]; encouragingly, a long-term cohort of high-risk D+/R− lung recipients managed with a proactive, multimodality prevention protocol achieved long-term survival comparable to lower-risk serostatus groups, suggesting that intensive, individualized prevention can offset serostatus-associated risk [88]. By contrast, in intermediate-risk R+ recipients, more intensive or prolonged prophylaxis has not been shown to improve graft or patient survival relative to preemptive approaches [83], and current evidence does not support routinely extending prophylaxis in this population solely to mitigate indirect effects [1]. Although prolonged prophylaxis effectively reduces CMV disease in high-risk recipients, its impact on indirect CMV-related outcomes remains less clearly established and must be balanced against drug toxicity (like myelosuppression, nephrotoxicity) cost, and the risk of antiviral resistance associated with extended exposure [1,79].

A more rational approach may lie in individualizing prevention according to a patient’s CMV-specific immune status rather than applying fixed durations. Immune-monitoring–guided strategies, in which prophylaxis duration is determined by recovery of CMV-specific cell-mediated immunity, can safely reduce antiviral exposure without increasing CMV infection, and may permit protective immune reconstitution to develop while still limiting early viral activity [89]. Such immune-guided and hybrid (prophylaxis-followed-by-preemptive) strategies represent the most promising direction for specifically mitigating the indirect effects of CMV and should be evaluated in trials that include indirect outcomes—rejection, graft survival, secondary infection, and mortality—as primary endpoints.

## 7. Conclusions

CMV should be regarded not only as a cause of direct post-transplant viral disease, but also as a major driver of indirect post-transplant morbidity. The evidence reviewed here indicates that CMV reshapes both innate and adaptive immunity, promotes endothelial and vascular injury, alters metabolic homeostasis, and impairs antimicrobial defense. Through these effects, CMV is linked to acute rejection, chronic allograft dysfunction, cardiovascular and thrombotic complications, post-transplant metabolic abnormalities, and secondary infections in solid organ transplant recipients. This interpretation is consistent with current international consensus guidelines, which recognize that CMV prevention improves outcomes not only by reducing overt CMV disease but also by mitigating its downstream indirect effects [1].

A central message of this review is that CMV acts as a persistent immunomodulatory pathogen rather than a simple marker of immunosuppression. Mechanistic studies support biologically plausible links between CMV and transplant injury, including monocyte reprogramming, NK-cell and T-cell dysregulation, altered antigen presentation, Th1/Th17-skewed inflammation, endothelial activation, and impaired pathogen recognition and clearance. At the same time, much of the available evidence remains observational, and for several outcomes it remains difficult to distinguish the direct contribution of CMV from the effects of overall immunosuppressive burden.

Several further limitations warrant emphasis. As noted, the confounding of CMV with overall immunosuppressive burden is difficult to resolve, since recipients who develop CMV replication are frequently those receiving more intensive immunosuppression and already at higher baseline risk of the relevant outcomes; few studies have been designed to separate these contributions. Heterogeneity compounds this problem: definitions of the exposure (CMV infection, DNAemia, or disease), diagnostic assays and viral-load thresholds, prevention and monitoring protocols, and outcome definitions vary widely between studies, limiting comparability and contributing to the conflicting findings noted throughout this review [2,51,52,81]. Much of the mechanistic detail, moreover, derives from in vitro systems and animal models that establish biological plausibility but may not faithfully reproduce human transplant biology, with direct confirmation in human allograft tissue often lacking [71]. Finally, transplant recipients—particularly kidney recipients—frequently have coexisting conditions, including diabetes, cardiovascular disease, chronic kidney disease, and polypharmacy, that independently contribute to the outcomes attributed to CMV [52,57]. These considerations do not negate the substantial evidence implicating CMV in indirect post-transplant morbidity, but they indicate that current conclusions should be regarded as associative and biologically plausible rather than definitive.

Despite growing evidence, important questions remain about the indirect effects of CMV and the most effective ways to mitigate them. Prospective studies with longer follow-up are needed to establish which indirect effects are truly CMV-mediated. Further research is also needed to determine whether current CMV prevention strategies are sufficient to limit these indirect consequences, whether universal prophylaxis or preemptive therapy is superior in specific clinical settings, and which subgroups of transplant recipients are most likely to derive benefit from one strategy over the other.

Both innate and adaptive immune mediators are necessary for control of CMV after transplantation [90,91,92,93]. Improved risk stratification based on CMV serostatus, transplant type, intensity of immunosuppression, and immune monitoring profiles may help identify patients in whom more intensive or individualized preventive approaches are warranted. In parallel, additional studies are needed to refine biomarkers of CMV-related immune dysregulation and endothelial injury. As emphasized in the broader literature on indirect CMV effects, future preventive strategies, including vaccine development, should be evaluated not only for their effect on direct CMV disease but also for their ability to reduce longer-term indirect outcomes [1,94].

In conclusion, recognition of the indirect effects of CMV should influence how CMV is studied, monitored, and prevented after transplantation. A more complete understanding of CMV-driven immune and vascular pathology may improve risk stratification and support more targeted approaches to prevention, ultimately reducing long-term graft injury and patient morbidity.

## Data Availability

No new data were created or analyzed in this study.

## Abbreviations

The following abbreviations are used in this manuscript:

ADMA	asymmetric dimethylarginine
BMI	body mass index
cGMP	cyclic guanosine monophosphate
CMV	cytomegalovirus
DNAemia	detectable viral DNA in blood
FMD	flow-mediated dilation
GAD65	glutamic acid decarboxylase 65
HbA1c	glycated hemoglobin A1c
HLA	human leukocyte antigen
HR	hazard ratio
ICAM-1	intercellular adhesion molecule 1
IFN-γ	interferon gamma
IL	interleukin
IRF3	interferon regulatory factor 3
MHC	major histocompatibility complex
NF-κB	nuclear factor kappa-light-chain-enhancer of activated B cells
NK	natural killer
OR	odds ratio
PAMPs	pathogen-associated molecular patterns
PBMCs	peripheral blood mononuclear cells
PJP	Pneumocystis jirovecii pneumonia
PTDM	post-transplant diabetes mellitus
RR	risk ratio
SOT	solid organ transplantation
STEMI	ST-elevation myocardial infarction
TCR	T-cell receptor
TCR-β	T-cell receptor beta chain
Th1	T helper type 1
Th17	T helper type 17
TLR	Toll-like receptor
TNF	tumor necrosis factor
TNF-α	tumor necrosis factor alpha
Treg	regulatory T cell
US28	CMV-encoded chemokine receptor US28
VCAM-1	vascular cell adhesion molecule 1
